# Flexible Thermo-Optic Variable Attenuator based on Long-Range Surface Plasmon-Polariton Waveguides

**DOI:** 10.3390/mi9080369

**Published:** 2018-07-26

**Authors:** Jie Tang, Yi-Ran Liu, Li-Jiang Zhang, Xing-Chang Fu, Xiao-Mei Xue, Guang Qian, Ning Zhao, Tong Zhang

**Affiliations:** 1 Key Laboratory of Micro-Inertial Instrument and Advanced Navigation Technology, Ministry of Education, and School of Instrument Science and Engineering, Southeast University, Nanjing 210096, China; tangjieck@126.com (J.T.); xiaomei_xue2011@163.com (X.-M.X.); chinaqgll@163.com (G.Q.); 2 Suzhou Key Laboratory of Metal Nano-Optoelectronic Technology, Suzhou Research Institute of Southeast University, Suzhou 215123, China; duduranla@163.com (Y.-R.L.); njzhao88@163.com (N.Z.); 3 Joint International Research Laboratory of Information Display and Visualization, School of Electronic Science and Engineering, Southeast University, Nanjing 210096, China; ljzhv@163.com (L.-J.Z.); pasf365@163.com (X.-C.F.)

**Keywords:** variable optical attenuator (VOA), surface plasmon-polariton (SPP), microwave photonics

## Abstract

A flexible thermo-optic variable attenuator based on long-range surface plasmon-polariton (LRSPP) waveguide for microwave photonic application was investigated. Low-loss polymer materials and high-quality silver strip were served as cladding layers and core layer of the LRSPP waveguide, respectively. By using finite element method (FEM), the thermal distribution and the optical field distribution have been carefully optimized. The fabricated device was characterized by end-fire excitation with a 1550 nm laser. The transmission performance of high-speed data and microwave modulated optical signal was measured while using a broadband microwave photonics link. The results indicated that the propagation loss of the LRSPP waveguide was about 1.92 dB/cm. The maximum attenuation of optical signal was about 28 dB at a driving voltage of 4.17 V, and the variable attenuation of microwave signals was obviously observed by applying different driving voltage to the heater. This flexible plasmonic variable attenuator is promising for chip-scale interconnection in high-density photonic integrated circuits and data transmission and amplitude control in microwave photonic systems.

## 1. Introduction

The flexible electronics and optical devices are promising for smart wearable devices, large capacity data communications, integrated optical sensing elements, and integrated optoelectronics [1,2,3]. Recently, the demands for high speed and large capacity interconnections in the optical communications and chip-to-chip signal transmissions increase rapidly. Flexible optical interconnection technology is an attractive solution for these applications, owing to the large bandwidth and the flexibility of the optical signals [4,5]. 

The variable optical attenuator (VOA) is an important optical component that is widely used in optical communication systems [6,7] and optical signal processing in microwave photonics [8,9]. Especially in microwave photonic systems, microwave signals are transmitted and processed in optical domain and the VOAs are always used to adjust and equalize the power of the optical signals in different parallel channels. With the development of integrated optics, many chip-scale integrated microwave photonic circuits that are used for signal processing and data transceiving have been realized [10,11]. In the future, large-scale multifunctional integrated optical systems will be implemented by using chip-to-chip hybrid interconnections. Hence, the VOAs with wide attenuation range, high-density integration, and flexible bending are necessary for parallel multi-channel optical interconnections.

In recent years, many kinds of VOAs with different structure and mechanism have been reported, such as radiation loss tuning in the S-bend waveguide [12], phase adjusting in Mach–Zehnder interferometers (MZIs) [13,14], optical evanescent field absorption in the waveguide [15], and light mode tuning in a multimode interference device (MMI) [16,17,18,19,20], etc. Although these works above present excellent performance in different aspects, such as low-power, low insertion loss, large attenuation range, but still not adequate to meet the requirements of sub-wavelength optical manipulation as well as transmission in higher integrated circuits in the future for the optical diffraction limit [12,21].

The surface plasmon-polariton (SPP), which is a transverse magnetic (TM) polarized plasmon mode that exists in the interface between a metal and a dielectric, has been shown to transmit optical signals beyond the diffraction limit [22,23,24]. It is very advantageous for high-density integrated optical systems [21]. With a symmetrical dielectric cladding and thin film core, the surface plasmon modes that are associated with the upper and lower of the metal-dielectric interfaces couple and form a low-loss symmetric mode, known as the long-range SPP (LRSPP) [25]. In comparison with the asymmetric short-range SPP (SRSPP) mode, the LRSPP mode has a large propagation length due to its low propagation loss and it can be applied to chip-to-chip interconnections. But, the SRSPP mode generally has a small mode size and it is suitable for compact integrated optic devices and hybrid integrated chips [26,27]. The SPP waveguides have been employed in various optical devices, such as VOAs [28], filters [29], switches [30], and modulators [31,32,33] for the refractive indices of the claddings can be adjusted by thermo-optic or electro-optic effect. The LRSPP waveguide consisting of dielectric claddings with similar refractive indices and the metal thin film is extremely sensitive to the symmetry of refractive indices. The mode cut-off of LRSPP in the metal strip can be acquired with an asymmetry in the refractive indices of the dielectric layers above and below the thin metal strip. For a slight asymmetry, the LRSPP mode is still supported, while if the asymmetry is too large the LRSPP mode becomes cut-off and no low loss modes are supported, resulting in asymmetric SRSPP mode with high propagation loss. Light input into the structure radiates away from the metal strip core. Besides, the mode size of the LRSPP waveguide can be adjusted by changing the thickness and width of the stripe core to obtain a low coupling loss with other kinds of waveguide devices or fibers.

Based on the sensitivity to interface property of the LRSPP waveguide mentioned above, several VOAs have been proposed and investigated. For example, a LRSPP VOA that is based on electro-optic controllable liquid crystals and polymers is investigated and it exhibits a good performance of low power, high extinction ratio, and low insertion loss [34]. For both polarization use, a plasmonic nanowire-based thermo-optic VOA with a cross-section of 250 nm × 250 nm shows a low polarization dependent loss of ±2.5 dB with a compact footprint of 1 mm [35]. Another thermo-optic LRSPP modulator with a length of 1 cm is investigated at telecom wavelengths and it shows low driving power and high extinction ratio [36]. However, most of these VOAs use metal strip core as the heater, which will limit the physical lifetime of the device because the thin metal strip core may be wrinkled and damaged at a high temperature.

In this paper, we design a flexible VOA with a structure consisting of LRSPP waveguide and a thermal heater. By using a simple spin-coating, photolithography, and wet etching process, the device has been fabricated and then characterized at a wavelength of 1.55 μm. The performance of optical attenuation and high speed data transmission are measured.

## 2. Device Design and Simulation

**Figure 1:**
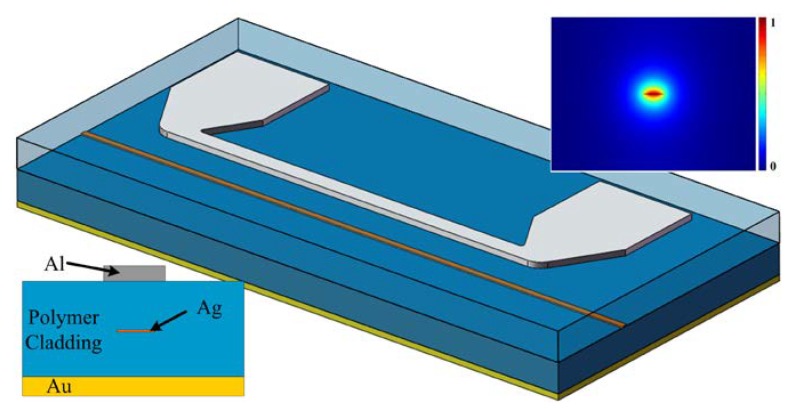
The schematic diagram of the variable optical attenuator (VOA). The bottom left inset is the cross-section and the top right inset is the optical field distribution of the long-range surface plasmon-polariton (LRSPP) waveguide.

The schematic diagram of the VOA based on LRSPP waveguide is shown in Figure 1. The bottom left inset is the cross-section of the device. It consists of a gold (Au) layer, lower polymer cladding, thin metal strip core, upper polymer cladding and a heater aligned on top of the upper cladding. The thicknesses of the upper and lower polymer cladding layers are both 15 µm. The silver (Ag) strip core of the LRSPP waveguide is carefully designed with an optimized parameter and the width and thickness is 4 μm and 12 nm, respectively. The heater is made of aluminum (Al). Its cross-section dimension is 10 μm wide and 150 nm thick and the length is 10 mm. The refractive indices of the polymer claddings are both 1.45 and that of the Ag strip core is 0.15 + 11.38i at 1550 nm. The calculated optical field distribution by the finite element method (FEM) is shown in the top right inset of Figure 1. It indicates that the optical field is symmetrically distributed in the interface between the claddings and the strip core, and most of the energy is distributed in the claddings next to the interface, which ensures a long-range propagation mode.

**Figure 2:**
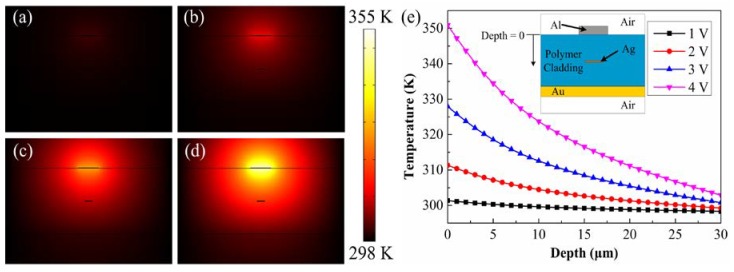
The calculated thermal distribution with applied voltage of (**a**) 1 V, (**b**) 2 V, (**c**) 3 V, (**d**) 4 V to the heater. (**e**) Temperature gradient in the depth of the device at different applied voltage.

The principle of the VOA is based on changing the radiation loss of the LRSPP waveguide resulting from the refractive index difference between the upper and lower claddings. When a voltage is applied to the heater, thermal energy being produced by resistive heating is transferred to the upper cladding, Ag strip, and lower cladding in turn. Then, a temperature gradient is established by the thermal energy and it increases with the applied voltage, as shown in Figure 2. The temperature gradient subsequently results in the refractive index difference between the upper and lower cladding due to the thermo-optic effect. Finally, the asymmetry of refractive indices results in the radiation loss of the LRSPP mode. Therefore, the optical output power of the device can be controlled by changing the temperature gradient by applying different voltages to the heater. The thermo-optic coefficient of the polymer is −1.758 × 10^−4^/°C. The negative thermo-optic coefficient results in the refractive index decrease with the increase in temperature.

According to the dimension of the heater, we can calculate resistance of the heater. By using FEM, we calculate the thermal distribution in the device while applying different voltages to the heater, as shown in Figure 2a–d. The corresponding temperature distribution is shown in Figure 2e. It shows a clear asymmetry distribution of the temperature in the upper and lower claddings when the voltage is applied. Temperature gradient also increases rapidly with the increasing applied voltage (see Figure 2e), which will result in a larger refractive index gradient and eventually enlarge the radiation loss of the LRSPP mode. Therefore, the propagation loss of the device can be adjusted by changing the applied voltage to the heater.

## 3. Experiments

**Figure 3:**
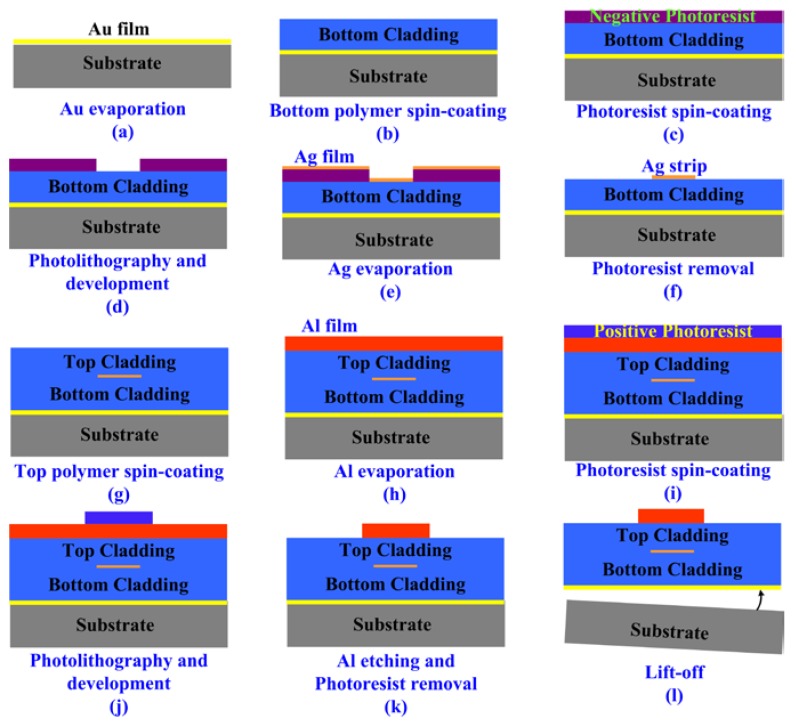
Fabrication process of the flexible LRSPP based VOA.

Figure 3 presents the fabrication process of the flexible thermo-optic VOA based on LRSPP waveguide. Firstly, a layer of 40 nm thick Au was thermally evaporated on the silicon wafer as an adhesion layer, which made it easy to peel off the flexible device from the wafer in the last step. Then, a layer of 15 µm thick ultraviolet (UV) cured epoxy resin was spin-coated on the Au layer as the lower polymer cladding. After a subsequent bake at 160 °C for 30 min, a layer of 2.5 μm thick negative photoresist was spin-coated and baked at 110 °C for 3 min. With a UV photolithography and development, the photoresist was patterned to fabricate a 4 µm wide strip groove. The electron beam evaporation method was used to deposit high-quality Ag thin metal film of 12 nm thick onto the negative photoresist layer. In the evaporation process, a high-purity (>99.999%) solid Ag and a very low deposit speed of 0.02 nm/s in a high vacuum of 1 × 10^−4^ Pa were used to ensure a high-quality Ag film. After that, the patterned negative photoresist layer was lift-off and a 4 µm wide Ag strip was left over to form the core layer of LRSPP waveguide. Then, another layer of 15 µm thick UV-cured epoxy resin was spin-coated and cured as upper cladding. To fabricate the heater, a layer of 150 nm Al film was thermally evaporated. Then, a layer of positive photoresist was spin-coated and patterned by UV photolithography. After that, wet etching was used to fabricate the Al heater with sodium hydroxide (NaOH) solution. Finally, the flexible device was lift-off from the silicon wafer. After the fabrication process above, the two end faces of the device were polished to minimize the coupling loss between the fiber arrays and the waveguide.

**Figure 4:**
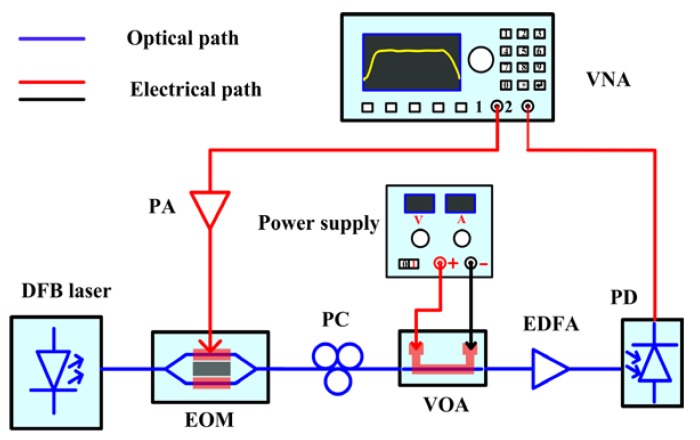
The measurement setup for the test of the optical attenuation and microwave transmission characteristics.

**Figure 5:**
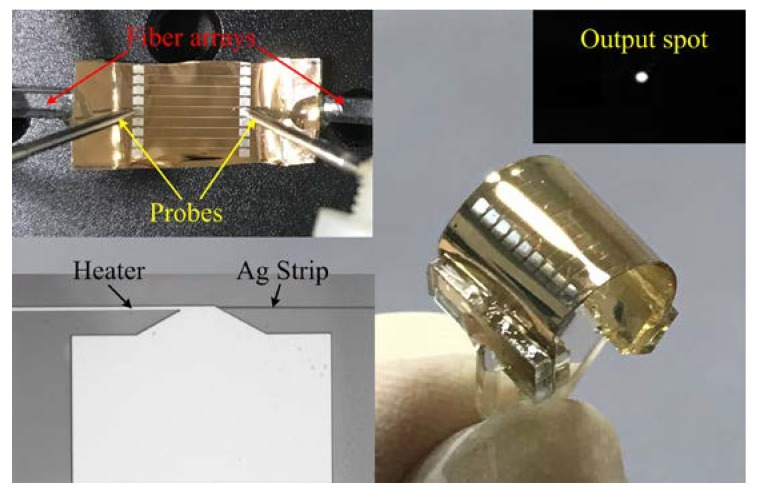
The photograph of the curved flexible thermo-optic VOA. Top left inset is the device under test and the driving voltage is applied through two probes. Top right inset is the near-field output spot of the LRSPP waveguide. Bottom left inset is the photograph taken by an optical microscope.

The schematic diagram of the measurement setup for the thermo-optic VOA is shown in Figure 4. To study the transmission characteristics of high frequency microwave signals, we constructed a broadband microwave photonics link with a bandwidth over 25 GHz. Vector network analyzer (VNA) with a bandwidth of 50 GHz was used to measure the transmission characteristics of the high frequency microwave signals. The light at 1550 nm from a distributed feedback (DFB) laser source was modulated by high frequency microwave signals via a lithium niobate (LiNbO_3_) electro-optic modulator (EOM). The modulated light was perpendicularly polarized to the waveguide plane by passing through a polarization controller (PC) and then coupled into the waveguide to excite LRSPP mode through a standard single mode fiber (SMF) array. To measure the optical attenuation characteristics of the VOA, a direct-current (DC) power supply was used to apply different voltages to the heater through two probes. In the output port of the device, a standard SMF array was used in order to couple the output light signal into an optical power meter to measure the propagation loss of the LRSPP waveguide and the optical attenuation characteristics of the VOA. Alternatively, the output light was also coupled into a broadband photodetector to demodulate the high frequency microwave signals. Herein, we used an Erbium-doped fiber amplifier (EDFA) with a gain of 20 dB to compensate for the insertion loss of the LRSPP waveguide-based VOA. Meanwhile, a microwave power amplifier (PA) with a gain of 19 dB was employed to compensate the total loss of the microwave photonic link. The fabricated thermo-optic VOA under test is shown in the top left inset of Figure 5.

## 4. Results and Discussion

**Figure 6:**
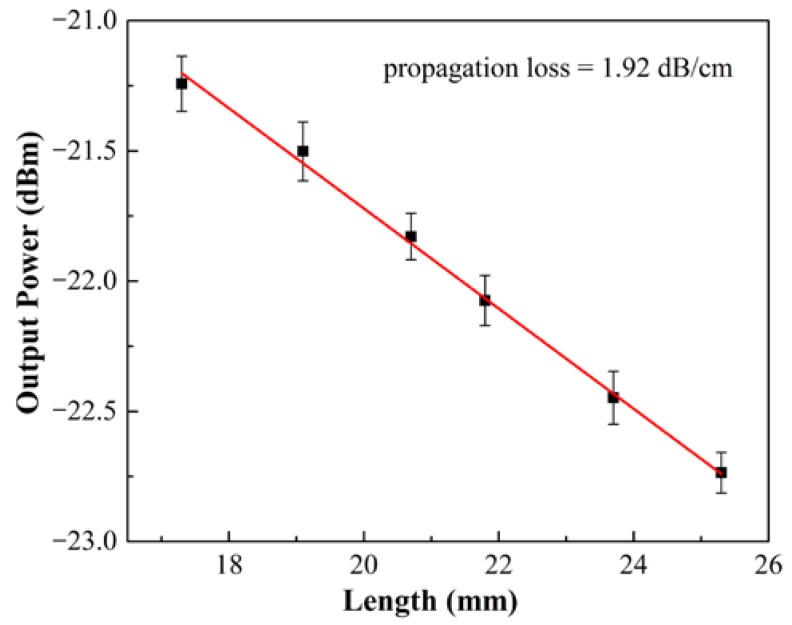
Measured propagation loss of the LRSPP waveguide by cut-back method.

**Figure 7:**
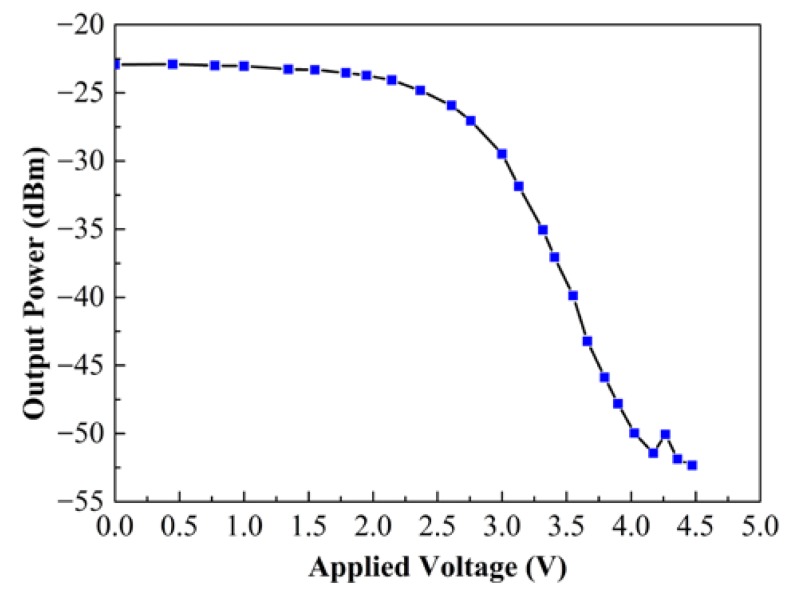
Measured optical output power of the thermo-optic VOA as a function of applied voltage to the heater.

The output light of LRSPP waveguide was observed before packing with the fiber array by using an infrared (IR) charge-coupled device (CCD). The near-field output spot of the device is shown in the top right inset of Figure 5. The bright spot shows that the fabricated LRSPP waveguide has a good performance in terms of light propagation. By coupling the output light into the optical power meter, the propagation loss of the LRSPP waveguide is measured at about 1.92 dB/cm by cut-back method, as shown in Figure 6. The results show that the propagation loss is at least two times lower than other previously reported flexible LRSPP waveguide [37,38]. It also indicates that the quality of the fabricated LRSPP waveguide is very high. In order to investigate the performance of the optical attenuation, the voltage is applied to the heater. As the applied voltage increases, the optical attenuation can be observed clearly, as shown in Figure 7. The measured results show that a maximum optical attenuation of over 28 dB is acquired at a driving voltage of 4.17 V. At the same time, we can see that the optical attenuation is inconspicuous when the applied voltage is lower than about 2.5 V. While the applied voltage is larger than 2.5 V, the output optical power decreases rapidly. The reason of this phenomenon can be attributed to the fact that the LRSPP mode still exists in spite of a slight asymmetry of the refractive index that is produced by a relatively low driving voltage. When a larger voltage is applied, the asymmetry is too large and the LRSPP mode becomes cut-off. In other words, the SPP mode changes from nonradiative mode to radiative mode in the case of a large asymmetry of the refractive index.

**Figure 8:**
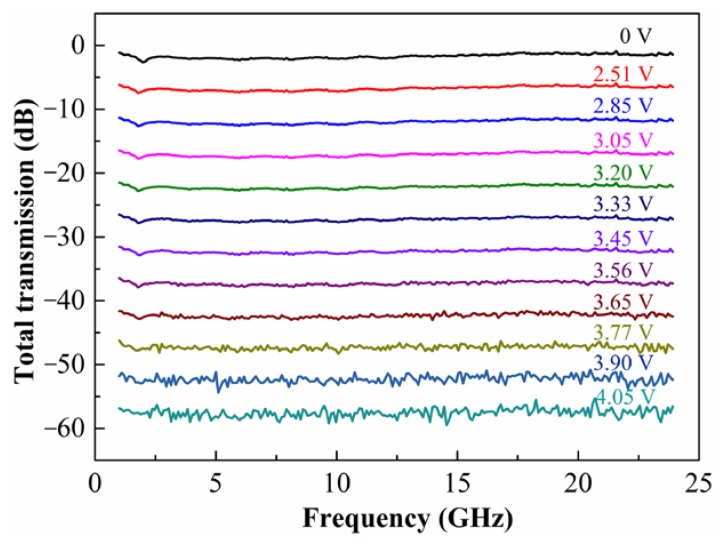
The total microwave transmission characteristics of the microwave photonics link at different applied voltage to the heater.

By using the microwave photonics link and the VNA, we can observe the transmission and attenuation characteristics of high frequency microwave signals. The microwave attenuation can be attributed to the decrease of the optical light coupled into the photodetector. According to the measured results of the optical attenuation above, the maximum optical attenuation of 28 dB can result in a maximum microwave attenuation of 56 dB for the square relation between optical loss and microwave loss. The measured microwave transmission and attenuation characteristics under different applied voltage are shown in Figure 8. In the experiment, we carefully adjust the voltage in order to acquire each microwave attenuation interval of 5 dB. Similar to the optical attenuation characteristics, the microwave attenuation increases with the increase of applied voltage. The microwave attenuation is also inconspicuous when the applied voltage is lower than about 2.5 V. Only 5 dB attenuation is acquired when the applied voltage increases from 0 V to 2.51 V. While the applied voltage is larger than 2.5 V, the microwave attenuation increases rapidly. Over 50 dB attenuation is acquired as the voltage increases from 2.51 V to 4.05 V. As the applied voltage increases, more noise appears in the transmission curve. The reason is that the optical power reached in the photodetector is too low to demodulate the high frequency microwave signals. In spite of the noise in the curve, a maximum attenuation of about 55 dB is acquired. 

**Figure 9:**
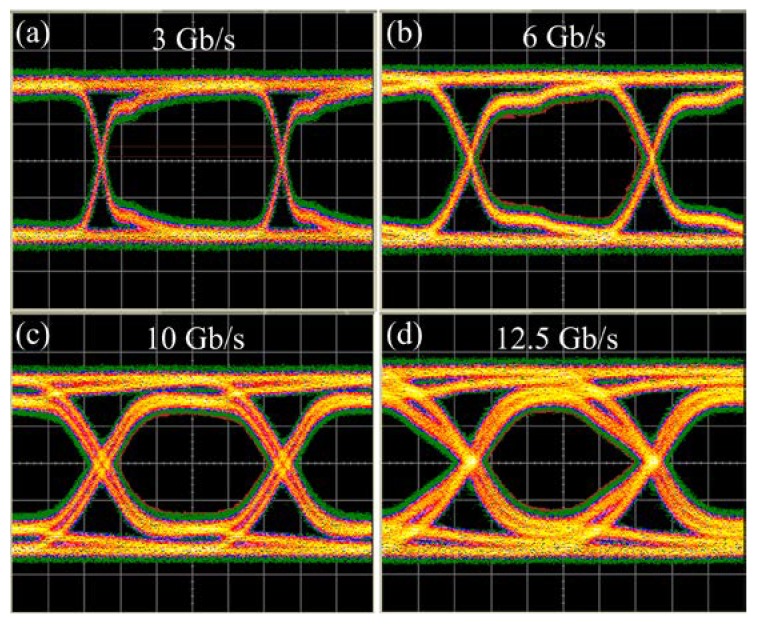
Measured eye diagrams of the total microwave photonics link at (**a**) 3 Gb/s, (**b**) 6 Gb/s, (**c**) 10 Gb/s, and (**d**) 12.5 Gb/s.

After the characterization of optical propagation loss and microwave transmission performance, we also carried out a data transmission experiment with the flexible LRSPP waveguide by using a bit-error-rate (BER) test system (N4903B, Agilent Technology, Santa Clara, CA, USA). Figure 9 shows the measured eye diagrams of the total microwave photonics link. A modulated light with 3 Gb/s, 6 Gb/s, 10 Gb/s, and 12.5 Gb/s pseudorandom-binary sequence (PRBS) (2^31^ − 1) generated by a pulse-pattern-generator (PPG) was transmitted through the flexible LRSPP waveguide. As shown in Figure 9, the eye is well open at a different transmission speed. The BER at 12.5 Gb/s was measured about 1.13 × 10^−10^. The results indicate that the flexible LRSPP waveguide is suitable for high speed optical interconnections.

Evidently, the fabricated devices still suffer from too high insertion loss to meet practical applications. In comparison with the other low power VOAs [39,40,41], the power consumption is a little larger as well. It is mainly due to the thick polymer cladding that extends the distance of thermal diffusion. However, a good performance of the optical and microwave attenuation has been acquired. Furthermore, the transmission of a light signal modulated by high frequency microwave signals through the flexible LRSPP waveguide-based VOA has been tested in spite of obstacles of large insertion loss, wave vector difference, and dispersion of the LRSPP mode. It is a potential solution to realize the flexible chip-scale interconnections and power equalization in the optical systems. The problem of large insertion loss can be solved by further optimizing the coupling loss between fiber and waveguide or using gain media doped polymer to reduce the propagation loss in the future.

## 5. Conclusions

We demonstrated a flexible thermo-optic variable attenuator based on LRSPP waveguide that can be used to transmit and equalize high frequency microwave signals in microwave photonic systems. The controllable optical attenuation has been realized by applied different driving voltage to the thermo-optic VOA. The experimental results show that the maximum optical attenuation of the VOA is over 28 dB at a driving voltage of 4.17 V. The maximum attenuation of microwave signals is about 55 dB. The flexible thermo-optic VOA using LRSPP waveguide is promising for chip-scale interconnection in high-density photonic integrated circuits and data transmission, amplitude control, and equalization in microwave photonic systems.
